# Supplementary material for: The interactome of intact mitochondria by cross-linking mass spectrometry provides evidence for coexisting respiratory supercomplexes
Source: Mol Cell Proteomics. 2017 Dec 8;17(2):216–32. doi: 10.1074/mcp.RA117.000470 (PMC5795388; doi:10.1074/mcp.RA117.000470)
Supplement: Supplemental Data [file supp_RA117.000470_133922_0_supp_23978_fzffwf.zip › spectra_annotation/mito_DR_spectra_annotation/30-1-4-1-4-1.pdf]

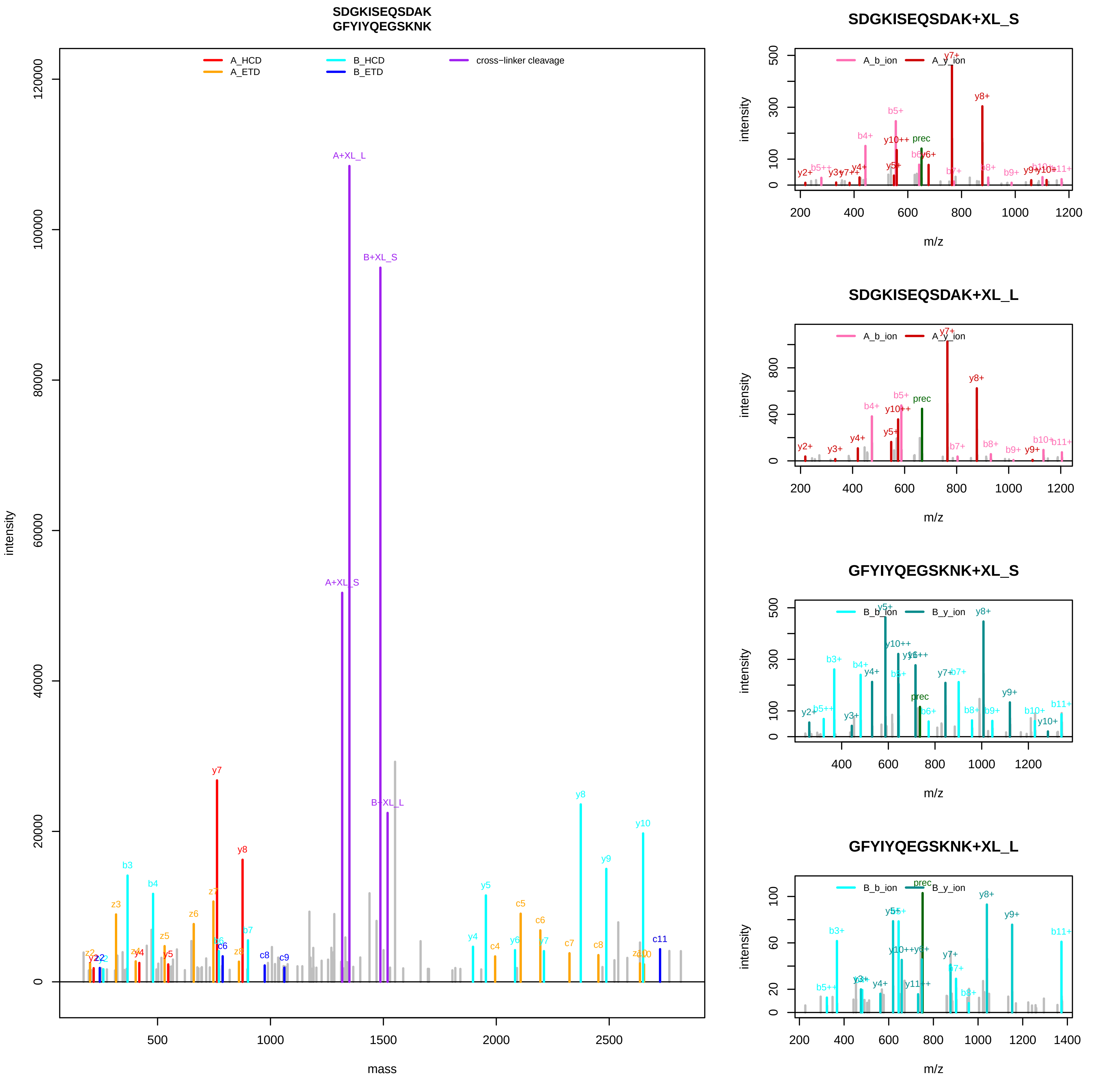

SDGKISEQSDAK
GFYIYQEGSKNK
A_HCD
A_ETD
B_HCD
B_ETD
cross−linker cleavage
intensity
mass
A+XL_L
B+XL_S
A+XL_S
B+XL_L
y7
y8
z3
b3
b4
z6
z7
z5
y5
b7
c8
c9
y4
y5
c4
c5
c6
y6
y7
c7
c8
y8
y9
y10
c11
SDGKISEQSDAK+XL_S
A_b_ion
A_y_ion
y7+
y8+
b5+
b4+
y10++
prec
m/z
SDGKISEQSDAK+XL_L
GFYIYQEGSKNK+XL_S
B_b_ion
B_y_ion
GFYIYQEGSKNK+XL_L
